# Establishment and Partial Characterization of Three Novel Permanent Cell Lines Originating from European Freshwater Fish Species

**DOI:** 10.3390/pathogens14060531

**Published:** 2025-05-26

**Authors:** Andor Doszpoly

**Affiliations:** HUN-REN Veterinary Medical Research Institute, 1143 Budapest, Hungary; doszpoly.andor@vmri.hun-ren.hu

**Keywords:** fish cell line, virus isolation, northern pike, European perch, asp

## Abstract

The establishment and partial characterization of three continuous cell lines from European freshwater fish species are provided. The three new cell lines, designated NPL-3, AF-1, and PF-1, were created from larvae of northern pike *(Esox lucius*) and fin tissues of asp (*Leuciscus aspius*) and European perch (*Perca fluviatilis*) fin tissues, respectively. All three cell lines have been subcultured more than 90 times since their establishment. Cells were optimally maintained at 25 °C in M199 medium supplemented with 10% fetal bovine serum. The NPL-3 and AF-1 cells are susceptible to spring viraemia of carp virus, pike fry rhabdovirus, ictalurid herpesvirus 2, and European catfish virus, while in the PF-1 cells, only the latter two viruses were successfully propagated. These newly established cell lines could serve as diagnostic tools for the aforementioned economically important viral diseases. They might be effective appliances for isolating novel viruses from northern pike, asp, European perch, and other closely related fish species.

## 1. Introduction

Aquaculture is the fastest-growing sector of agriculture in the world. The annual production of fish farming has overtaken the capture production recently (FAO; https://www.fao.org/fishery/en/fishstat, accessed on 1 March 2025). As fish farming continues to increase worldwide, the related research areas, for example, fish virology, are also needed to adapt to the novel challenges. In the ages of metagenomics, the discovery of novel viruses is mainly achieved by next-generation sequencing (NGS). However, for understanding the biology of the viruses, and fighting against them with vaccines, and antiviral materials, the isolation and propagation of these viruses are still essential. Permanent cell lines are useful biological monitoring tools for viral diseases and besides the primary cell cultures the only tool for isolating emerging viruses, and then propagating them for further studies.

A few examples from the last couple of years are as follows: the dwarf gourami (*Trichogaster lalius*) cell line has been established and proved to be highly susceptible to megalocytiviruses and rhabdoviruses, making it an excellent tool for the diagnosis, prevention, and control of viral diseases in ornamental fish [1]. With the recently developed Chinese bahaba (*Bahaba taipingensis*) and Chinese perch (*Siniperca chuatsi*) cell lines, the interactions between immune mechanisms and megalocytiviruses and rhabdoviruses have been studied, respectively [2,3]. The cultivation of Cyprinid herpesvirus 2 was difficult in widely used cell lines, such as EPC (Epithelioma Papulosum Cyprini), FHM (Fathead Minnow), and KF-1 (Koi Fin), because these cell lines did not support its continuous propagation after the 5th passage [4]. However, this obstacle has been overcome by establishing of a novel cell line from goldfish (*Carassius auratus*) [5]. Other areas of virology need cell lines as well; for example, Atlantic salmon (*Salmo salar*) cardiac cells have been created for in vitro host–pathogen interaction modelling and pathogenesis for cardiotropic viruses (Infectious salmon anaemia virus and Salmonid alphavirus 1) [6]. Cell lines are also essential for the development and production of attenuated and inactivated vaccines. Modern recombinant vaccine candidates have been developed against Cyprinid herpesvirus 3 (koi herpesvirus) and Anguillid herpesvirus 1 in CCB (Common Carp Brain) and EK-1 (Eel Kidney) cell lines [7,8]. Apart from the use of fish cell lines in virology, other key aspects of in vitro research should be mentioned, such as fish growth, reproduction, genetics, biotechnology, toxicology, metabolism, transgenic fish production, and biobanking [9]. Although many permanent fish cell lines were established in the last 60 years, the majority of them are from saltwater fish species or fish species endemic to Asia or America [10,11,12]. Moreover, the majority of the permanent cell lines from freshwater fish species originate from a few economically important fish, for example, rainbow trout (*Oncorhynchus mykiss*), goldfish, and snakeheads (family Channidae) [11]. Only a very small portion of the freshwater fish cell lines are available commercially, for example, EPC, RTG (Rainbow Trout Gonad), and KF-1. In recent years, the establishment of permanent fish cell lines from fish species native to Central Europe has been commenced in our lab. In the present study, the successful development and partial characterization of three cell lines originating from asp (*Leuciscus aspius*), northern pike (*Esox lucius*), and European perch (*Perca fluviatilis*) is reported. All of these species are considered excellent gamefish; moreover, the pike and the perch are commonly bred in aquaculture for human consumption.

## 2. Materials and Methods

### 2.1. Establishment of the Primary Cells

Fin tissues were used from the asp and perch (both were app. 5 cm long). In case of the northern pike, 1-day old whole fry were utilized. Fish were stunned by destroying the spinal cord behind the head. Tissues were disinfected by dipping into 70% ethanol, then they were cut into small pieces by using a sharp scissors (1–2 mm^3^), and rinsed nine times for 2–3 min in MEM medium (Biosera, Nuaillé, France) containing penicillin (500 units/mL)-streptomycin (500 µg/mL)-amphotericin B (1.25 µg/mL) (Lonza, Walkersville, MD) followed by a long rinse (2 h) at room temperature. Subsequently, the tissue pieces were digested by approximately 10-fold volume cell culture grade 0.25% trypsin solution (Biosera, Nuaillé, France) in a shaker for 15 min at 25 °C with 180 rpm, then supernatant was collected (trypsin activity was inhibited by adding 10% fetal bovine serum (FBS)) and fresh trypsin was added and the digestion was repeated. Cells were centrifuged at 100 g for 10 min, then supernatant was discarded and the cells were resuspended in a small defined volume of a cell culture medium. Counting the cells in a haemocytometer (Luna-IITM Automated Cell Counter (Logos Biosystem, Anyang, South Korea)) using 1% trypan blue dye was performed, then the concentration was adjusted to 600.000 cells/mL with cell culture medium in 25 cm^2^ cell culture flasks (Cell+ for challenging adherent cells, Sarstedt, Germany, Nümbrecht). Mediums were supplemented with 20% FBS (Biosera, Nuaillé, France), 1% HEPES buffer (1M), and 2% penicillin-streptomycin-amphotericin B and incubated at 20 and 25 °C. The medium was changed daily on the cells in the first week. MEM, DMEM, L-15, and 199 media (Biosera, Nuaillé, France) were used. L-15 medium has been designed for cultivating cells without CO_2_ equilibration; it has been used in several studies developing fish cell lines [1,2,13]. MEM and DMEM cell culture media have been widely used in cellular studies and experiments due to their versatility and compatibility with a wide range of cell types; they have been used for important fish cell lines, such as KF-1 [14], EPC [15], WSS-2 (White Sturgeon Spleen) [16]. Medium 199 was originally developed for nutritional studies of chick embryo fibroblasts, but it has broad species applicability, particularly for the cultivation of non-transformed cells [3,17,18].

### 2.2. Subcultivation and Storage of the Cells

First passage of the cells was carried out after the cells formed a 100% monolayer in a 1:2 ratio. Later serial passages were conducted in 7–10 days intervals; the split ratio was gradually increased from 1:2 to 1:6. An amount of 20% FBS supplemented medium was used until the 10th passage, then it was reduced to 10%. The cells were sub-cultured continuously at an interval of 7–10 days and attained the passage level of 90 over a period of 2 years. Cells were cryopreserved at different passage levels (5, 10, 20, 30, etc.) in their own medium containing 10% dimethyl sulfoxide (DMSO) and 10% FBS. Cryovials were placed into isopropanol container and were put into −80 °C freezer for 24 h, then cryovials were transferred to liquid nitrogen tank. For investigating the short-term storage of the cells, flasks with 100% confluent monolayers were transferred and kept at 15 °C for 3 months. One day before the subcultivation, fresh medium was added and the cells were moved back to 25 °C.

### 2.3. Growth Characteristics Investigations

The examination of the effect of the incubation temperature on cell growth was carried out as follows: cells were seeded in 25 cm^2^ cell culture flasks and incubated at 25 °C for four h, then the flasks were moved to 15 °C, 20 °C, 25 °C, and 30 °C. The temperature range was selected according to the natural water temperatures of the springs and summers of the region. The number of the living cells was counted by a haemocytometer (using 1% trypan blue) at each temperature daily from duplicate flasks for one week. These experiments were performed when the passage numbers of the cell lines were between 40 and 50.

### 2.4. Molecular Investigations

Partial amplification and sequencing of a mitochondrial gene (cytochrome c oxidase subunit I, COI) and an approximately 900 bp long fragment of the 28S ribosomal DNA (rDNA) were performed on each cell line in order to proof their origin [19,20]. DNA was extracted from the cells (at the passage number 40) by the Viral Nucleic Acid Extraction Kit II (Geneaid Biotech Ltd., New Taipei City, Taiwan) according to the instructions of the manufacturer. The PCR reaction mixture contained 25 µL PrimeSTAR^®^ MAX DNA Polymerase (Takara Bio Inc., Kusatsu, Japan), 1-1 µL forward and reverse primer (10 pmol/µL) [19], 21 µL nuclease-free water, and 2 µL DNA template. For the rDNA amplification, the following mixture was used: 34 µL distilled water, 10 µL of 5 × HF Green buffer (Phusion, Thermo Fisher Scientific, Waltham, MA, USA), 0.5 µL Phusion enzyme (Thermo Fisher Scientific, Waltham, MA, USA), 1 µL (10 pmol/µL) of each primer [20], 1.5 µL of dNTP solution of 10 mM concentration, and 2 µL target DNA. The thermal cycler conditions were performed as follows: 1 cycle at 98 °C for 3 min, followed by 45 cycles at 98 °C for 10 s, 55 °C (54 °C) for 30 s, 72 °C for 1 min, with a final elongation at 72 °C for 7 min. The PCR products were sequenced using a BigDye Terminator v3.1 Cycle Sequencing Kit (Thermo Fisher Scientific, Waltham, MA, USA), and the electrophoresis was carried out on an ABI Prism 3100 Genetic Analyzer (Thermo Fisher Scientific, Waltham, MA, USA) by a commercial service provider. The DNA sequences were analyzed with BioEdit [21] software package (version 7.2.5), and the sequences were identified with the BLASTn algorithm at the NCBI portal.

### 2.5. Viral Susceptibility Assays

Virus inoculation was carried out with strains of four economically important virus species affecting the aquaculture. The European catfish virus (ECV) strain was isolated from brown bullhead (*Ameiurus nebulosus*) [22,23]. The ictalurid herpesvirus 2 (IcHV-2) strain was isolated in Italy from black bullhead (*Ameiurus melas*) [24,25]. The spring viraemia of carp virus (SVCV) was isolated in our lab [26], while the pike fry rhabdovirus (PFRV) was kindly provided by Dr. Tamás Juhász. Cells were seeded into 25 cm^2^ cell culture flasks (25 °C) and 24 h later virus suspensions (10 µL) were added. The viruses’ TCID_50_/mL values were the following: PFRV: 1 × 10^7^, SVCV: 1.78 × 10^7^, IcHV-2: 1 × 10^6^ and ECV: 5.62 × 10^7^. The cells inoculated with SVCV and PFRV were incubated at 20 °C while the cells with ECV and IcHV-2 stayed at 25 °C. Cells were monitored daily for the signs of cytopathic effects (CPE). Images were taken by Axio Observer D1 (Zeiss, Oberkochen, Germany) microscope.

### 2.6. Titration of the Viruses

Titration of the viruses was also carried out in each cell line. Cells were seeded on 96-well plates and incubated at 25 °C. Next day 10-fold serial dilution of the viruses were added and were incubated for 10 days at 20 and 25 °C. TCID_50_/mL values were determined using the method by Reed and Muench [27]. The virus titers on the established cell lines were compared to that of on the EPC cell line (ATCC CRL-2872) using two-tailed Student’s *t*-test. *p*-values of 0.05 were considered to be significant.

## 3. Results

### 3.1. Establishment of the Cell Lines

Primary cell cultures were created successfully from northern pike larvae (NPL-3), asp (AF-1), and European perch (PF-1) fin tissues. In all cases, the monolayers became complete in 5–7 days. In the beginning, all three cell lines consisted of fibroblastic and epithelial-like cells. Subsequently, the cells were subcultured at a 1:2 ratio which was gradually raised to 1:6. Also, after the first passages, the cells were moved from the Cell+ flasks (for challenging adherent cells) to normal 25 cm^2^ flasks, and the FBS concentration was halved from 20% to 10% after the 10th passage. The morphology of the cells has changed from the early mixed stage to predominantly fibroblast-like cells in AF-1 and NPL-3, while the PF-1 consisted mainly of epithelial-like cells.

After three month of storage at 15 °C, the average viability of the cells proved to be around 60% measured by the automated cell counter in the case of all three cell lines, while cell viability was around 90–95% after one year-long storage following recovery from liquid nitrogen. No morphological changes or alterations in growth speed were observed after the cryopreservation. All cell lines were passaged 90 times.

### 3.2. Growth Characteristics

**Figure 1:**
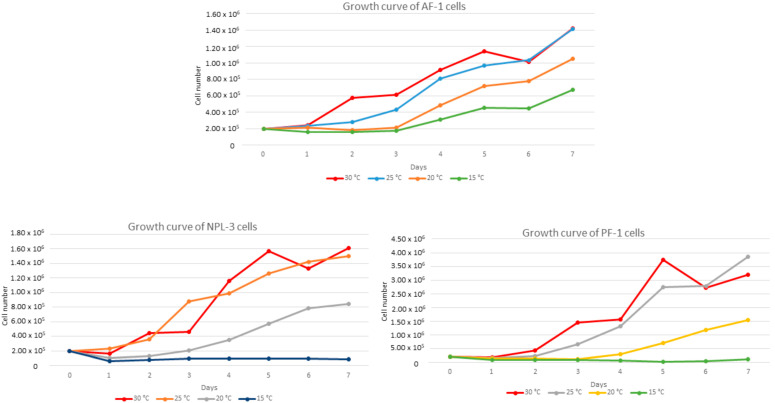
Growth characteristics of the three established cell lines (AF-1, NPL-3, and PF-1) at different temperatures; cell number was represented as mean (±SEM).

Cell proliferation was studied at a temperature range between 15 °C and 30 °C with 5 °C intervals. Preliminary studies showed that cells died at 37 °C and they did not grow at 10 °C. It seems that the 25 °C was optimal for all three cell lines (Figure 1). Although they grew a bit more rapidly at 30 °C, the cell aging was also a bit accelerated. After one week, the number of floating dead cells was larger.

### 3.3. Molecular Study

The sequence analysis (BLASTn) of the approximately 1500 and 900 bp long fragments of the mitochondrial gene (COI) and the 28S rDNA confirmed that the AF-1, NPL-3, and PF-1 cell lines undoubtedly originated from asp, northern pike, and European perch, respectively.

### 3.4. Cell Line Susceptibility Assays and Virus Titrations

**Figure 2:**
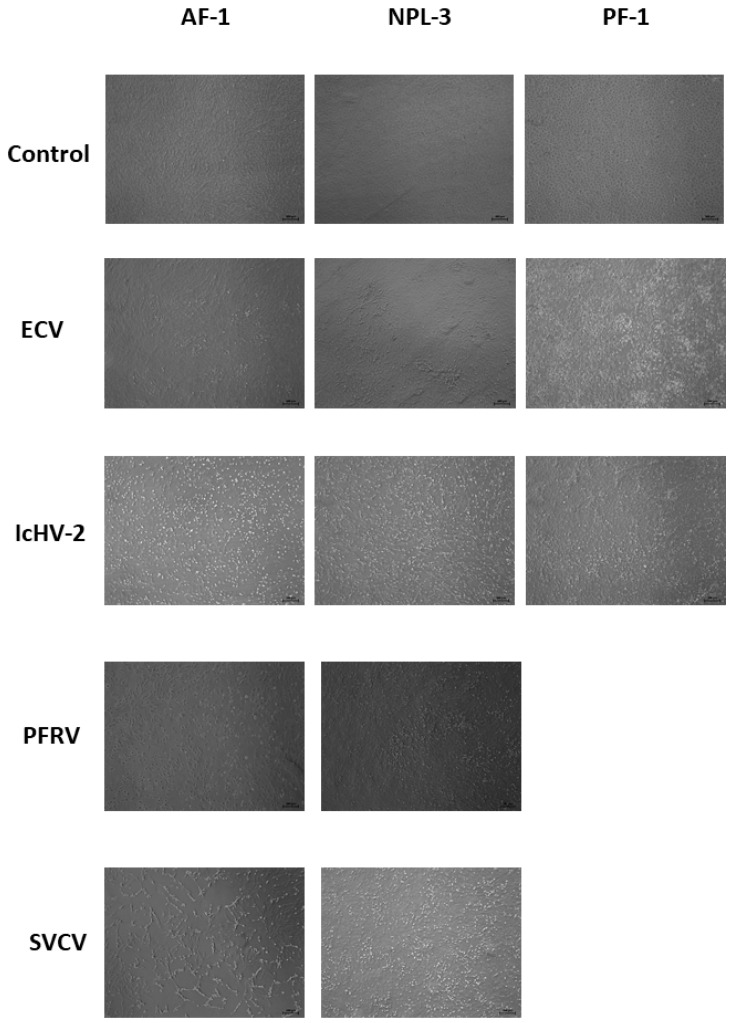
Cytopathogenic effect caused by the different viruses (ECV, IcHV-2, PFRV, SVCV) in the established cell lines (AF-1, NPL-3, PF-1) (50× magnitude). Cells were photographed at 4 days post inoculation.

In the viral inoculation assays, all tested viruses (ECV, IcHV-2, SVCV, and PFRV) caused cytopathic effect (CPE) in NPL-3 and AF-1 monolayers (Figure 2). However, PF-1 cells proved to be refractory to rhabdoviruses (SVCV and PFRV), and CPE was observed only in cells infected with ECV and IcHV-2 (Figure 2).

TCID_50_/mL of the different viruses were determined in the susceptible cell lines. EPC cell line was used as a control. Plates were read after 10 days of inoculation (Table 1). In the case of the rhabdoviruses (PFRV, SVCV), the novel cell lines did not prove to be more susceptible to the viruses than EPC. Although PFRV had higher titer in AF-1 cells, the difference was not statistically significant. For the propagation of ECV, NPL-3 and PF-1 proved to be better than EPC. The titer in the NPL-3 cells was 10x higher, which proved to be a statistically significant difference. As for the IcHV-2, NPL-3 and AF-1 produced higher titers, but statistical calculations did not show significant differences.

## 4. Discussion

Over the past 60 years, hundreds of fish cell lines have been established. According to the first comprehensive review on this subject, 60 permanent fish cell lines had already been developed until 1980 [12], while the last review, published a few years ago, reported a total of 783 cell lines derived from various tissues of different fish species [11]. The majority of these cell lines were created for virological purposes, including studies on viral aetiology and pathology, host–pathogen interactions, virus diagnosis, and vaccine development. Other applications include research in cytogenetics, carcinogenesis, cellular physiology, toxicology, and transgenics [11]. In general, cell culture systems are considered effective alternatives to the use of live animals in biological research. Emerging applications for fish cell lines include cell-based food production, conservation and biobanking, and regenerative medicine [28,29,30].

In the present study, three permanent cell lines were successfully established from three European fish species: northern pike, asp, and European perch. To date, no commercially available permanent cell lines exist from these fish species. From these species, only one ovarian cell line from northern pike was developed and reported more than four decades ago [31]. All three novel cell lines showed optimal growth in medium 199 at 25 °C. This medium has previously been demonstrated to be effective in the establishment of fish cell lines; for example, the SSF-2 (fin) and SSO-2 (internal organs) cells, derived from Siberian sturgeon (*Acipenser baerii*), have been cultivated in medium 199 [17].

In terms of viral susceptibility of the novel cell lines, the AF-1 and NPL-3 cell lines were permissive to all four fish viruses tested in this study, whereas the PF-1 line was only susceptible to ECV and IcHV-2. For comparison, we have only one data point; the earlier established northern pike cell line [31] was also shown to be susceptible to SVCV [12]. Each of the viruses examined in this study poses a significant threat to aquaculture and belongs to distinct viral families. ECV is a highly pathogenic member of the family *Iridoviridae*; it affects several fish species, including wels catfish (*Silurus glanis*) [32,33], black and brown bullhead (*Ameiurus melas* and *A. nebulosus*) [22,23,34,35], turbot (*Scophthalmus maximus*) [36], and pike-perch (*Sander lucioperca*) [37]. A closely related virus to ECV, the epizootic haematopoietic necrosis virus (EHNV) has been described in European perch in Australia and has demonstrated high viral load [38]. Propagating the ECV in PF-1 cells produced high titer as well (Table 1).

IcHV-2, a double-stranded DNA virus from the family *Alloherpesviridae*, is also of concern due to its capacity to cause up to 100% mortality in black bullhead [24]. PFRV and SVCV, both members of the family *Rhabdoviridae* (single-stranded RNA viruses), are known to inflict significant losses in carp and northern pike farming [39].

The World Organisation for Animal Health (WOAH, formerly OIE) currently recommends the use of EPC and FHM (Fat Head Minnow) cell lines for the isolation of SVCV and EHNV (ECV). The novel cell lines established in this study proved to be highly permissive to several of these viruses, thereby offering valuable tools for future virological research. While the EPC cell line remains the most widely used and versatile for fish virology—supporting the propagation of all aforementioned viruses—our findings suggest that the new cell lines, particularly NPL-3, may provide enhanced performance for specific viruses. Indeed, ECV titres in NPL-3 were tenfold higher than those in EPC, and this difference was statistically significant. NPL-3 also showed high sensitivity to IcHV-2; however, the observed increase in viral titre was not statistically significant due to large standard deviations. Nonetheless, these findings suggest that NPL-3 may serve as an effective platform for virus isolation.

In virology, virus isolation in cell cultures is considered the gold standard for virus identification. Therefore, the establishment of cell lines from novel fish species or from novel tissues is crucial, especially for viruses that have been detected but not yet isolated. Similar to viruses of higher vertebrates, many fish viruses exhibit a narrow host range or strict host specificity. Several of these fish pathogens are capable of causing severe outbreaks with high mortality rates. For instance, koi herpesvirus (KHV) can only be propagated in specific koi cell lines (e.g., KF-1). There remain several important fish viruses that have never been successfully isolated in vitro, such as carp oedema virus (CEV), complicating comprehensive studies, including vaccine development, due to the inability to generate sufficient viral particles for in vivo challenges. As for the fish species used in this study for establishing cell lines, northern pike herpesvirus has been detected several times by PCR or electron microscopy [40] but it has not been isolated yet; retroviruses potentially associated with lymphosarcoma in northern pike were also reported but not isolated [40,41]; perch herpesvirus [42] and cyprinid herpesvirus 5 in asp [43] have been detected only by PCR. A circovirus has also been identified in asp by PCR; however, no correlation between viral presence and disease has yet been demonstrated [44]. Although perch rhabdovirus (*Perhabdovirus*) has been successfully isolated in the RTG cell line, the yield of the propagated virus has not been enough for further characterization [45]. With the successful establishment of these new cell lines, the possibility is given for further research into these poorly characterized fish viruses and novel emerging ones as well.

## 5. Conclusions

Three permanent cell lines were established and partially characterized from three European freshwater fish species (asp, European perch, and northern pike). All cell lines proved to be permissive to two economically important fish viruses (ECV and IcHV-2); moreover, NPL-3 and AF-1 were susceptible to two fish rhabdoviruses (PFRV and SVCV) as well, offering valuable tools for future virological research and diagnostics. These novel cell lines fill a gap, as no commercially available permanent lines previously existed from these fish species. Compared to generally used cell lines like EPC, one of the new lines, the NPL-3, may offer enhanced viral propagation for ECV. Moreover, these cell lines provide promising tools for studying viruses that have not yet been isolated, enabling a deeper understanding of host–pathogen interactions and emerging threats in aquaculture.

## Figures and Tables

**Table 1:** Mean TCID_50_/mL of the viruses in four cell lines. Values higher than the control (EPC) are in bold, while lower values are in italics. Statistically significant values are marked with *.

	SVCV	PFRV	ECV	IcHV-2
EPC	7.81 × 10^7^	1.05 × 10^7^	3.16 × 10^5^	1.39 × 10^5^
AF-1	1.86 × 10^6^ *	**1.63 × 10^7^**	4.39 × 10^4^ *	**4.39 × 10^5^**
NPL-3	6.58 × 10^6^ *	*7.81 × 10^5^*	3.16 × 10^6^ *	**1.86 × 10^7^**
PF-1	-	-	**4.39 × 10^5^**	*4.39 × 10^4^*

## Data Availability

The data that support the findings of this study are available from the corresponding author upon reasonable request.
